# Describing unpaid carers’ health service use in local areas across Wales: A retrospective cohort study using linked routinely collected data

**DOI:** 10.23889/ijpds.v8i2.2251

**Published:** 2023-09-14

**Authors:** Laura Bentley, Jerlyn Peh, Georgia Beckett-Hill, Karen Hodgson, Ashley Akbari, Claire Newman, Owen Davies, Walid Chehtane, Lisa Trigg, Joanna Dundon, Gareth John, Alisha Davies

**Affiliations:** 1 Public Health Wales, Cardiff, United Kingdom; 2 Population Data Science, Swansea University Medical School, Faculty of Medicine, Health & Life Science, Swansea University, Swansea, United Kingdom; 3 Digital Health and Care Wales, Cardiff, United Kingdom

## Objectives

Using anonymised linked data across primary care general practice (GP) and local authority (LA) services to (1) identify unpaid carers in Swansea and Neath Port Talbot (NPT), (2) describe their health and health service use and, (3) compare these with a matched non-carer population.

## Method

Unpaid carers were identified using a) LA carers’ assessment data and b) GP Read codes within the Secured Anonymised Information Linkage (SAIL) Databank. An age, sex and area-matched non-carers cohort was created using demographic data and assigned pseudo-index dates. Linked GP and secondary care data was used to establish GP interactions, hospital admissions, emergency department and outpatient attendances in the year prior to identification as a carer. Long-term conditions (LTCs) were identified using published Cambridge multimorbidity Read code lists. Chi-square, Mann Whitney U-test, and rate ratios were used to test differences in aforementioned factors between carers and non-carers.

## Results

We have identified a total of 2,950 unpaid carers (N=2,024 in NPT; N=926 in Swansea), primarily via Read codes (80% in NPT; 70% in Swansea). Overlap between LA and GP identified individuals is less than five percent, and GP identified individuals are significantly younger than LA identified (NPT: χ2=176, p<0.001; Swansea: χ2=35.0, p<0.001). Further research is currently ongoing to utilise these anonymised linked data to ascertain key differences between carers and non-carers in the two local authorities. Results will include the significance of differences in rates of GP interactions, emergency department attendances, hospital admissions, outpatient attendances, rates of multimorbidity (0, 1, 2+ conditions), and top five specific LTCs between carers and matched non-carers in NPT and Swansea.

## Conclusion

We demonstrate the novel use of local authority-held data linked to national anonymised data sources to provide locally informative evidence for this priority population. Results will provide novel insight into the health and health service usage of unpaid carers at a LA level, assisting evidence-informed local support for unpaid carers.

